# A pilot study of the Leicester ED medical infrared imaging protocol in fever and sepsis

**DOI:** 10.1371/journal.pone.0201562

**Published:** 2018-07-31

**Authors:** Timothy J. Coats, Mohamed Morsy, Sana Naseer, Karoly Keresztes, Sarina Hussain, Katie Dexter, Mark R. Sims

**Affiliations:** 1 Department of Cardiovascular Sciences, Emergency Medicine Academic Group, University of Leicester, Leicester, United Kingdom; 2 Department of Anaesthesia and Intensive Care, Faculty of medicine, Minia University, Minia, Egypt; 3 Department of Physics and Astronomy, University of Leicester, Leicester, United Kingdom; Azienda Ospedaliero Universitaria Careggi, ITALY

## Abstract

**Background:**

Medical Infrared Imaging (MII) is an investigative method that can be potentially used in emergency care to non-invasively detect thermal signatures associated with change in blood flow. We have developed a protocol for the use of MII in the Emergency Department (ED) and shown that it is feasible. To derive initial data for sample size calculations, we performed an exploratory study in patients with fever and sepsis.

**Methods:**

The Leicester MII protocol was used to image the temperature patterns along the arm among three patient groups (control, fever and sepsis) of a total 56 patients. Anatomical markers were used to divide this gradient into upper arm, forearm, hand and finger regions. Variations in measurements within and between these regions were described.

**Results:**

The thermal gradient down the arm was successfully extracted in all patients. The distribution of values in each region of the arm was described in control, fever and sepsis patients. There was a significant gradient between upper arm and finger in controls (2.75, p < 0.0001), but no gradient in fever (p = 0.944) or sepsis (p = 0.710). This was reflected in the finger/arm difference, which was of -2.74°C (±3.50) in controls, -0.39C (±2.48) in fever, and -1.80°C (±3.09) in sepsis.

**Conclusions:**

This study found different thermal gradients along the arm in control and febrile groups, and defined the degree of individual variation. It is likely that the difference between upper arm temperature and finger temperature (representing the temperature gradient down the arm) may be more useful than absolute measurements in future studies.

## Introduction

Thermography in patients with infections has been in widespread use in mass screening programs to prevent travellers transmitting infectious disease [1–3], and to provide an initial screen for animal [4,5] and humans [6] suspected to have significant infections. However there are significant methodological problems [7] and the evidence of effectiveness of fever screening as a public health measure is weak [6,8].

Medical infrared imaging has potential uses in emergency care as many acute presentations change blood flow and hence also change thermal patterns. There is some experimental animal evidence of ability to detect pneumothorax [9], and emergency care clinical studies have looked at detection of fractures (10), and compartment syndrome [11]. These few clinical emergency care studies have poor designs and no standardised methodology [10,12]. We have previously described the development of the Leicester Emergency Department Medical Infrared Imaging Protocol (Leicester ED MII Protocol)[13], which gives standardisation within the practical constraints of thermal imaging in an ED environment.

It is a common clinical observation that peripheral temperature varies in sepsis, with patients first undergoing a peripheral warm phase (due to vasodilatation–‘warm shock’) followed by increasingly cold peripheries as increasing systematic vascular resistance. There is animal evidence of ‘centralisation’ (cold peripheries) defined by thermal imaging in a pig model of systemic inflammatory response [14]. There has been no study of the diagnostic utility of Medical Infrared Imaging (MII) in fever and sepsis in emergency care. In order to obtain initial information on which to base the sample size calculation for a formal diagnostic study we tested the feasibility of data acquisition, processing and analysis using the Leicester ED MII Protocol in patients presenting with fever.

## Methods

Ethical approval was granted by East Midlands—Nottingham 2 Research Ethics committee and NHS Research and Development (R&D) approval was also granted. Good Clinical Practice (GCP) training and consent training courses were undertaken by all involved in study implementation.

As this was a feasibility study a formal sample size calculation could not be carried out. A convenience sample of patents was recruited as part of an undergraduate research project and the rationale for the imaging methods have been previously published [15]. Control patients were defined as presenting with any complaint (not affecting the upper limb) and with normal temperature. Study patients all had a suspected infectious cause for fever, and were divided into two groups. The Fever group was defined as a tympanic temperature reading >37.5°C without any features of sepsis, and the Sepsis group was defined by meeting at least two Systemic Inflammatory Response Syndrome (SIRS) criteria according to the 2012 definition of sepsis [16].

A FLIR T650sc camera [17] was used (thermal sensitivity of <0.034 °C at 30°C). Each imaging session was carried out in the patient’s bay in the ED and lasted about 20 minutes. Acclimatisation of the camera components to room temperature was achieved by switching the camera on 2 hours prior to imaging. Suitable patients were approached and informed consent was obtained. The patient’s most available arm (free from medical intervention such as a blood pressure cuff or cannula) was selected for imaging. Patients were asked to expose this arm, either by changing into a hospital gown or by removal of outer clothing. Once the arm was exposed a 5–10 minute acclimatisation period was given during which anatomical land markers were attached [13].

Background thermal noise was blocked using a plain white bedsheet hanging vertically behind the patient. The patient was asked to hold an arm out horizontally in front of the sheet. The camera was manually focused and an image of the anterior surface of the arm was acquired. Images were stored directly onto the FLIR camera labelled with study ID, local temperature, and local humidity levels.

Customised software was written which normalised the images, subtract the background noise and identified the midline of the anterior surface of the arm [18]. The data from each pixel touched by the midline was extracted from the image to give a single thermal gradient down the arm. The anatomical markers were used to divide this gradient into upper arm, forearm, hand and finger regions. The D’Agostino & Pearson normality test was used to assess the pattern of temperature distribution within each region, and the mean temperature for the whole arm and for each region was calculated and used in further analysis (this method accounted for different arm length between individuals).

The mean and standard deviation of temperature for the whole arm and each arm region was calculated for the three patient groups (control, fever and sepsis). The temperature patterns along the arm are compared using a one-way ANOVA with a post-hoc comparison. The difference between the upper arm and the finger temperature was calculated for each individual, and the mean and standard deviation calculated for each group.

## Results

56 patients were imaged (35 control, 16 fever and five sepsis patients). Most patients had the right arm imaged (clinical examination takes place from the patient’s right, so ED cubicles are designed to leave the right side of the body unobstructed). Thermal images were successfully acquired and the thermal gradient down the arm was successfully extracted in all patients. The shape of the distribution of temperatures was tested and there was no evidence on non-normality.

**Fig 1:**
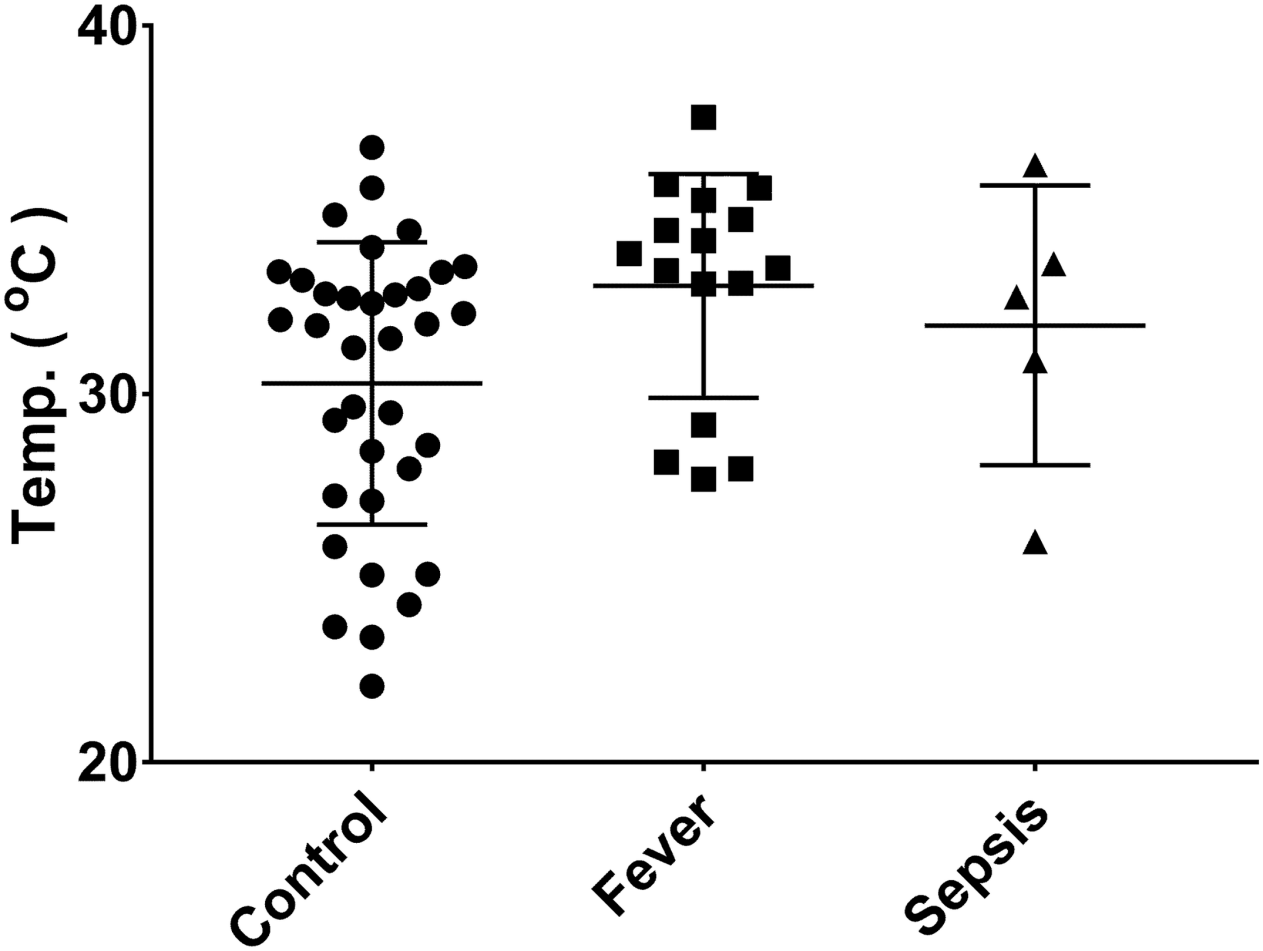
Distribution of individual readings in ‘finger’ region in control, fever and sepsis patients.

**Fig 2:**
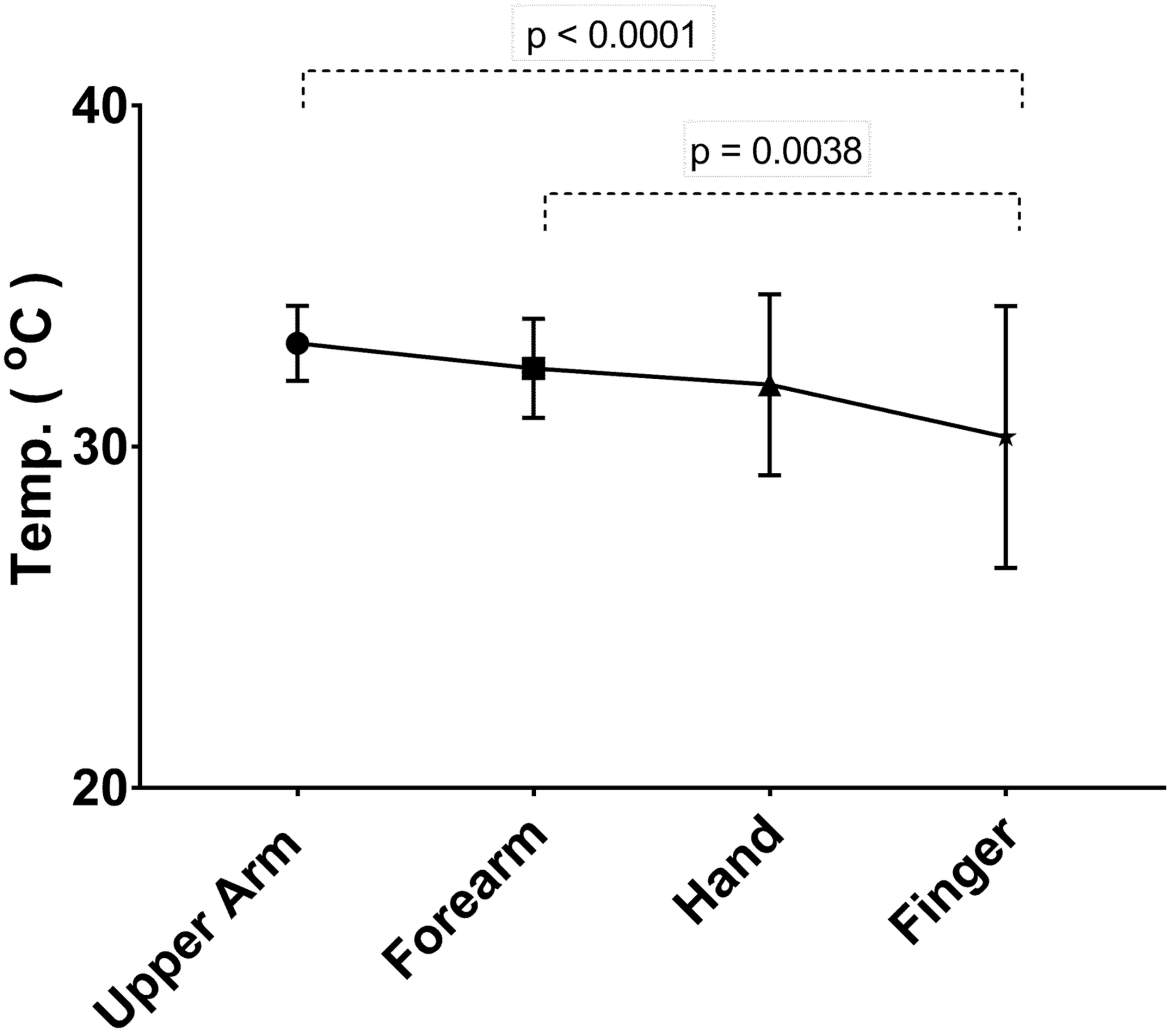
Temperature gradient in control group (mean and standard deviation) showing a significant decrease along the arm.

**Fig 3:**
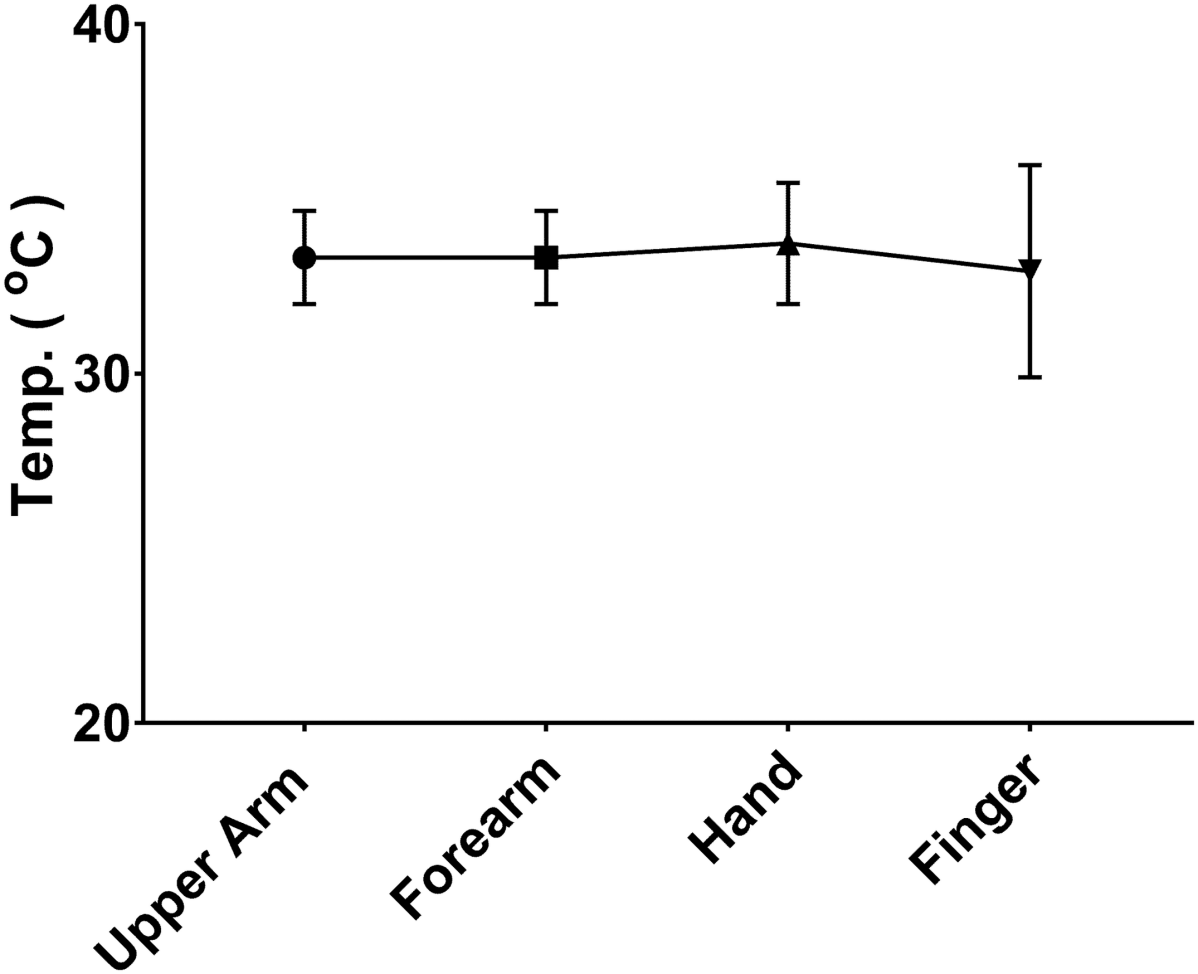
Temperature gradient in Fever group (mean and standard deviation) showing no significant change along the arm.

The temperature in each arm region (mean and standard deviation) is shown in Table 1. There was most variation in the finger region (Fig 1), with a trend to a bimodal distribution in fever and sepsis. There was no statistically significant difference in the mean temperature of whole arm (p = 0.477). However, there was a change in the pattern along the arm. In the control group there was a significant temperature drop along the arm of 2.75°C, from 33.03°C in the upper arm to 30.28°C on the fingers (p<0.0001) (Fig 2), whereas in the fever group there was no drop in temperature along the arm (p = 0.944) (Fig 3). There was also no drop in temperature in sepsis (p = 0.710), although there was only 5 patients in this group so confidence intervals were wide. In all groups there was progressively increasing variation (wider distributions) along the arm (Table 1).

**Table 1:** Temperature for whole arm and each region of the arm.

	Whole arm	Upper arm	Forearm	Hand	Fingers
**Control**	32.33±1.54	33.03±.09	33.30±1.45	33.81±2.65	30.28±3.83
**Fever**	33.51±1.28	33.32±1.33	33.32±1.33	33.72±1.73	32.93±3.03
**Sepsis**	33.32±2.24	33.66±1.42	33.46±1.98	33.52±2.81	31.86±3.79

**Fig 4:**
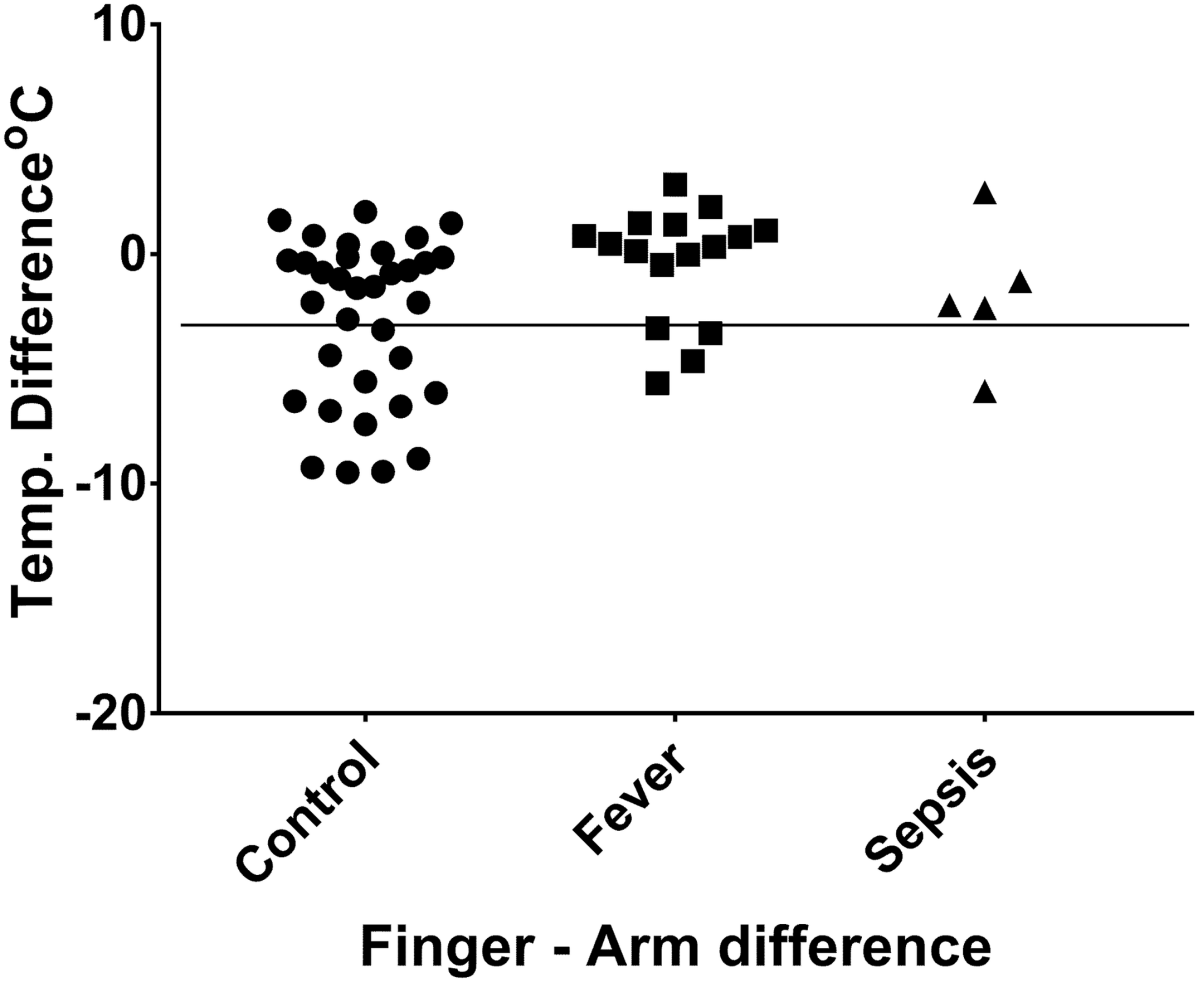
Difference between upper arm and finger temperature for each patient.

When the difference between the finger and upper arm temperatures was calculated (with a negative difference meaning colder fingers) the control group had a difference of -2.74°C (±3.50), the fever group had a difference of -0.39C (±2.48), and the sepsis group had a difference of -1.80°C (±3.09) (Fig 4).

On visual inspection of the images it was noticed that 3 of the patients among fever group showed a ‘blotchy’ pattern over the upper arm and forearm which looked like the same appearance (in the thermal spectrum) as the mottling seen in hypoperfused babies (in the visual spectrum). This novel finding was not detected in any control group patient.

## Discussion

For centuries the change in temperature of the extremities has been used by physicians as a crude form of assessing peripheral perfusion when assessing sick patients. The results presented here quantify the same findings in a more objective manner.

The Leicester MII Protocol is a novel methodology which does not conform to the current recognised standards for medical thermal imaging, as these standards would be impossible to apply in an emergency care environment. The Leicester MII Protocol can be criticised for not controlling all environmental variables, however whether or not the Protocol can be used is a practical rather than a theoretical question, which will only be resolved by a diagnostic study. In this paper we lay the foundation for a future study by successfully demonstrating the feasibility of a novel method of thermal data acquisition and processing in Emergency Care for patients with fever and sepsis. The results give data for temperature variations in different regions of the arm which can be used as a basis for future sample size calculations.

In all groups we found that there is more variation in temperature distally, which fits with previous thermography data in healthy controls [19], and with clinical experience. There was a clear equalisation of peripheral (fingers and hands) and more central (upper arm) temperature in patients with fever, which probably reflects the peripheral vasodilatation associated with fever.

It is difficult to draw firm conclusions about patients with sepsis, as there were too few in this group. However, the data did not show any clear-cut difference between the fever and sepsis groups. This could be due to the somewhat artificial current distinction between fever and sepsis (2 or more SIRS criteria) that may not sufficiently take account of individual variation in vasomotor and cardiovascular compensation for the effects of infection. In fever and sepsis there was (on visual inspection) some indication of a binary distribution, with some individuals having warm fingers and some cool. This tentative observation warrants further investigation in a large patient group. The definition of ‘sepsis’ is a current hot topic for debate and it may be that this type of novel measurement of functional changes in peripheral circulation could add to patient evaluation.

When the images were reviewed medical infrared imaging identified a mottling effect in a number of fever patients that was absent in all control patients. Mottling is often visible to the human eye in children and critically ill adults, indicating inadequate skin perfusion and vasomotor instability [20], but his effect is not seen in the visible spectrum in ED patients. Quantitative analysis of ‘mottling’ in images is a challenge, but like other forms of medical imaging (such as X-rays) a skilled human observer can recognise patterns. Thermal mottling needs further evaluation before any comment can be made on its utility, at present it is simply an interesting observation.

This study also discovered a novel limitation to the standard methods of analysing thermal images. The standard definition of ‘regions of interest’ are polygons drawn to cover areas of the body, the edges of which are defined by surface markings such as skin creases [21]. We followed this principle, although taking a line of readings down the midline of the arm rather than drawing a polygon. In the arm the boundaries between regions (such as the skin crease in the middle of the antecubital fossa) also coincide with ‘hotspots’ on the thermal image caused by the proximity of blood vessels to the skin surface. This means that a small change in the position of the boundary will make a large change to the average temperature in the adjacent regions. From this study we wonder if the definition of ‘regions of interest’ for medical thermal imaging protocols may need to define the regions by the anatomy of heat distribution around the body rather than by surface anatomy. This is a novel methodological issue which needs to be resolved for future work.

The small size of the sepsis group was a significant limitation of this study. Sepsis patients were particularly difficult to recruit due to severity of illness and time constraints of an emergency care environment, and had a lower prevalence than anticipated. The current method of data extraction and analysis is labour intensive, so the technique is limited to research studies. However, if future studies show utility there is the potential for automation of the process.

## Conclusion

The results of the patient study suggest that different thermal gradients along the arm can be defined in control and febrile patients. It is likely that the difference between upper arm temperature and finger temperature (temperature gradient down the arm) may be more useful in future studies than absolute measurements.
